# Experimental Measurements of Mechanical Properties of PUR Foam Used for Testing Medical Devices and Instruments Depending on Temperature, Density and Strain Rate

**DOI:** 10.3390/ma13204560

**Published:** 2020-10-14

**Authors:** Zdenek Horak, Karel Dvorak, Lucie Zarybnicka, Hana Vojackova, Jana Dvorakova, Miloslav Vilimek

**Affiliations:** 1 Department of Technical Studies, College of Polytechnics Jihlava, Tolsteho 16, 58601 Jihlava, Czech Republic; karel.dvorak@vspj.cz (K.D.); lucie.zarybnicka@vspj.cz (L.Z.); hana.vojackova@vspj.cz (H.V.); jana.dvorakova@vspj.cz (J.D.); 2 Institute of Theoretical and Applied Mechanics of the Czech Academy of Sciences, Centre Telc, Prosecka 809/76, 19000 Prague, Czech Republic; 3 Department of Mechanics, Biomechanics and Mechatronics, Faculty of Mechanical Engineering, Czech Technical University in Prague, Technicka 4, 16607 Prague, Czech Republic; miloslav.vilimek@fs.cvut.cz

**Keywords:** mechanical properties, polyurethane foam, rigid polyurethane foam, thermal loading, temperature

## Abstract

Rigid polyurethane (PUR) foam is products used as a biomedical material for medical device testing. Thermal stability is a very important parameter for evaluating the feasibility of use for testing surgical instrument load during drilling. This work aimed to perform experimental measurements to determine the dependence of the mechanical properties of a certified PUR on temperature, strain rate and density. Experimental measurements were realised for three types of the PUR samples with different density 10, 25 and 40 pounds per cubic foot. The samples were characterised in terms of their mechanical properties evaluated from tensile and compression tests at temperatures of 25 °C, 90 °C and 155 °C. Furthermore, the structures of the samples were characterised using optical microscope, their thermal properties were characterised by thermogravimetric analysis, and their density and stiffness with the effect of temperature was monitored. The results show that it is optimal not only for mechanical testing but also for testing surgical instruments that generate heat during machining. On the basis of experimental measurements and evaluations of the obtained values, the tested materials are suitable for mechanical testing of medical devices. At the same time, this material is also suitable for testing surgical instruments that generate heat during machining.

## 1. Introduction

Rigid polyurethane (PUR) foam is one of the most popular products among polymeric foams used for biomedical applications [1,2,3]. PUR is a thermoset produced by polyaddition polymerization of diisocyanates and polyols [4]. They are crosslinked polymers completed with specific additives according to the final use of the polymer, i.e., pigments, fillers (calcium carbonate, aluminium hydroxide, silica, kaolin, etc.), flame retardants and foam stabiliser [5,6]. Thermal stability of PUR depends on the type of urethane group. It is in the range from 120 to 250 °C. The glass transition temperature for these materials is around 77–116 °C [7].

Solid PUR foam (Sawbones, USA) that provides a uniform structure with only ±10% variation in density according to standard ASTM F-1839-08 is, in practice, used as a standard certified material for testing orthopaedic devices and instruments and, therefore, was used in the present study. The material properties of PUR foam are similar to those of human bone [8,9], and it is an alternative material to human cadaver bone for testing and demonstrating orthopaedic implants, instruments and instrumentation [10,11,12]. The use of PUR foam as an artificial biomedical material for testing of medical devices is advantageous from ethical, hygienic and processing perspectives. Manufacturers of tools used in operations such as drills, cutters, saws and saw blades also use this specific material for experimental wear testing of these tools and for verifying their service life. A critical factor that is generated during machining is a large amount of heat, which, however, fundamentally degrades bone tissue. In clinical practice, the degradation of bone tissue by heat generated during the drilling of holes in the bone in preparation holes for bone screws or implants is a research topic of great interest [13,14,15]. Heat damage to bone tissue affects healing and implant fixation into bone and, in extreme cases, can lead to medical device loosening. Thus, surgical instrument manufacturers are constantly developing new tools using structural materials in conjunction with special cutting edge geometries and special coatings with a single goal: reducing the amount of heat generated during drilling. The wear rate, damage and lifetime of all of these tools are subsequently tested using PUR foam.

The mechanical properties of polymeric materials are well known to be substantially affected by their temperature [16,17] and strain rate [18,19,20]. Concerning their wide range of applications and knowledge of their weaknesses, the mechanical properties of PUR foam have been a subject of continuous research because its poor mechanical properties limit its practice use. A literature review reveals that PUR foam has been tested by thermogravimetric analysis (TGA) [16,17,21,22] and that its mechanical properties in tension [23,24,25], compression [9,26,27,28,29] and fatigue [30] have been determined. However, the mechanical properties of PUR foam have not yet been analysed with respect to their dependence on temperature, strain rate and density. Therefore, the testing of surgical drills and rigid milling cutters for PUR foam certified according to ASTM F-1839-08 has not been objectively proven to not yield distorted results that differ from experimental measurements, possibly leading to incorrect conclusions.

In the present work, we carried out experimental measurements with the objective of determining how the mechanical properties of PUR foam vary with temperature, strain rate and density. The subject of testing was PUR foams with densities of 10, 25 and 40 PCFs (pounds per cubic foot). PUR foams with densities 5–8 PCF correspond to synthetic thoracic vertebrae (spongious bone) [31]. A density of about 20 PCF can be found for the femur (spongious bone), and a density of 40 PCF is generelly close to the cortial bone [32]. Furthermore, the objective was to verify experimentally the thermal stability of rigid polyurethane foam in the temperature range corresponding to the temperature load during drilling and to verify the feasibility of using PUR foam for testing surgical instruments for clinical use. PUR foam is used to test the wear of cutting tools. However, heat is generated during machining, which can change the PUR foam’s mechanical properties and thus reduce the wear rate of the tested cutting tools. The change in the PUR foam’s mechanical properties due to heat action can affect the validity of the experimental tests performed in this way. The mechanical properties of samples were characterised using tensile and compression tests, their structure was characterised by digital optical microscopy and their thermal properties were characterised by TGA. Manufacturers of surgical instruments develop a new design for tool cutting geometry using finite element method (FEM). The key to their implementation is a detailed knowledge of PUR foam’s material parameters depending on the temperature, density and strain rate. The outputs of the performed experimental measurements can be therefore used as inputs for numerical simulations.

## 2. Materials and Methods

### 2.1. Materials

Standardised rigid polyurethane (PUR) foam blocks (Sawbones Europe AB, Malmö, SWE) with uniform structure and ±10% variation in density were used for all analyses. The closed-cell foam conformed to standard ASTM F-1839-08. The density of the used PUR foam was in full accordance with standard ASTM D1622-08. Experimental measurements were carried out on three different densities of PUR foams—10, 25 and 40 PCF (pounds per cubic foot); the physical properties of the materials used in the experiments are described in Table 1. The samples were stored in a desiccator containing silica gel to prevent moisture absorption, which could affect subsequent testing. The test material was stored for a long time at a constant temperature of 22 °C and relative humidity of 40%.

### 2.2. Surface Roughness Measurement

To determine the roughness of the analysed samples, optical imaging measurements were performed using a VHX-6000 digital microscope (Keyence, Osaka, Japan). This device used optical triangulation and has a resolution of approximately 1 µm. The surface roughness parameters were determined according to ISO 25178. Six samples were analyzed for each PUR foam density, with one unmachined area on each. For each surface, estimates were derived from three scan repetitions, which were used to estimate the surface roughness (maximum height $S_{z}$, arithmetical mean height $S_{a}$ and root mean square height $S_{q}$). The dimensions of the analysed PUR foam samples with different densities (10, 25 and 40 PCF) were 15 × 15 × 5 mm.

### 2.3. Thermogravimetric Analysis

Thermal decomposition of PUR foams was determined using an STA 504 thermal analyser (TA Instruments, Wetzlar, Germany). This method was used for real-time measurements of weight loss of the examined materials as a function of temperature. Measurements were performed under a nitrogen atmosphere at a heating rate of 20 °C/min in the temperature range 20–460 °C. The producer guarantees the quality standard of all PUR foam; therefore, the TGA analysis was performed on only one sample. Samples were cut into small rectangular pieces weighing approximately 5–10 mg using a sharp razor blade. Before experimental measurements, samples were conditioned for 24 h at a constant temperature of 22 °C and relative humidity of 40%.

### 2.4. Tension Tests

**Figure 1:**
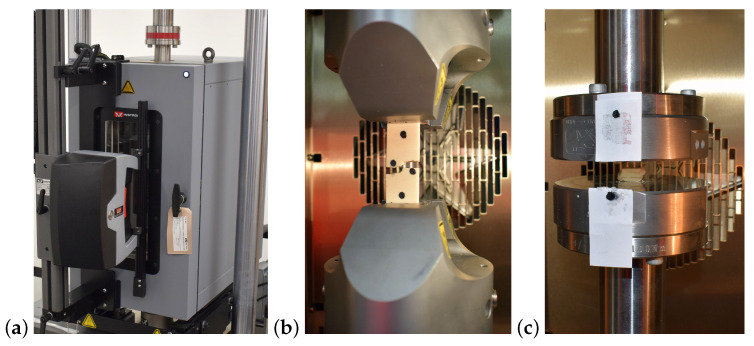
(**a**) Testing setup with heating chamber and video extensometer, (**b**) a test sample for the tension test, and (**c**) a test sample for the compression test.

Tensile tests were performed on an ElectroPuls E10000 electric test instrument (Instron, High Wycombe, UK) designed for dynamic and static testing and equipped with a heating chamber and video extensometer (Figure 1a,b). Seven samples with dimensions of 20 × 80 × 5 mm were tested for each density group of the analysed PUR foam. Specimens were loaded quasi-statically until failure under two different strain rates, v=4.5 and 45 mm/min, and at three different temperatures: 25 °C, 90 °C and 155 °C. Before the start of the test, all samples were conditioned in heating chamber for 5 min at the temperature at which the experimental tests were carried out. The samples were clamped in flat jaws, and the free sample length was 50 mm. Two markers were placed on the sample for measuring axial deformation at a distance of 30 mm. Deformation characteristics were measured using a video extensometer to detect the change of the marker position in the axial directions. Load–displacement data were recorded with a frequency of 10 Hz during the experiment. Tensile modulus was defined as the slope of the straight-line portion of a stress–strain curve. The tensile strength of the material was defined as the maximum amount of tensile stress that it could withstand before failure.

### 2.5. Compression Tests

Compression tests were performed on an ElectroPuls E10000 electric test instrument (Instron, High Wycombe, UK) equipped with a heating chamber Figure 1c. Seven samples with dimensions of 10 × 10 × 15 mm were tested for each group of analysed PUR foam. Specimens were loaded quasi-statically to a maximum compression force of 7 kN under two different strain rates, v=4.5 and 45 mm/min, and for three different temperatures: 25 °C, 90 °C and 155 °C. Before the start of the test, all samples were conditioned in a heating chamber for 5 min at the temperature at which the experimental tests were carried out. Specimen displacements were recorded from the crosshead movement. Load–displacement data were recorded with a frequency of 10 Hz during the experiment. The compressive modulus was defined as the slope of the straight-line portion of the stress–strain curve. Compressive strength was defined as the maximum stress a material could withstand under crush loading.

## 3. Results

### 3.1. Surface Roughness Measurement

From the experimental measurements, the three physical surface roughness parameters were analysed. $S_{z}$ is defined as the sum of the largest peak height values and the largest pit depth value within a defined area. $S_{a}$ is defined as the extension of the arithmetical mean height of a line to a surface. This parameter is generally used to evaluate surface roughness and is defined as

(1) $$S_{a}=\frac{1}{A}\;\int \int \;\left|Z\left(x,y\right)\right|\;dxdy.$$

$S_{q}$ represents the root mean square value of ordinate values within the definition area. It is equivalent to the standard deviation of heights defined as

(2) $$S_{q}=\sqrt{\frac{1}{A}\;\int \int \;Z^{2}\left(x,y\right)\;dxdy}.$$

The experimentally measured surface roughness parameters are given in Table 2. The highest value of all parameters was set for the sample with the lowest density, PCF 10 ($S_{a}=0.093$ mm, $S_{z}=0.933$ mm and $S_{q}=0.115$ mm). The lowest value of all parameters was set for the sample with the highest density, PCF 40 ($S_{a}=0.048$ mm, $S_{z}=0.647$ mm and $S_{q}=0.063$ mm). Surface roughness is clearly strongly dependent on the sample density and volume fraction.

The results corresponded to the theoretical idea: samples with the lowest density exhibited the worst quality of surface because content pores in their structure [33]. The results of the measured roughnesses also have an effect on the results of the mechanical properties. The higher the value of roughness, the easier it was to material manufacture, the lower the thermal load during the drilling process and the less thermally stressed the material. Of course, the material also showed lower strength, so the results of mechanical testing were worse for material with density PFC 10.

**Figure 2:**
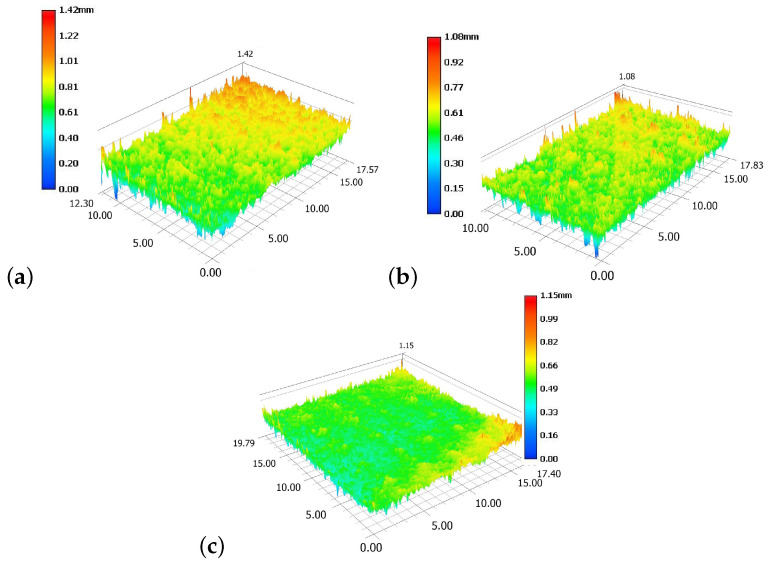
Surface analysis for all types of samples: PUR foam pounds per cubic foot (PCF) 10 (**a**), PUR foam PCF 25 (**b**), and PUR foam PCF 40 (**c**).

The results correlate with the observation that, with increasing density of the samples, their surface roughness decreases. The PFC 10 samples contain larger pores in their structure than the PFC 40 sample (see Figure 2). This effect strongly influences their mechanical properties. Samples with a higher density appear more compatible; better mechanical testing is therefore expected.

### 3.2. Thermogravimetric Analysis

**Figure 3:**
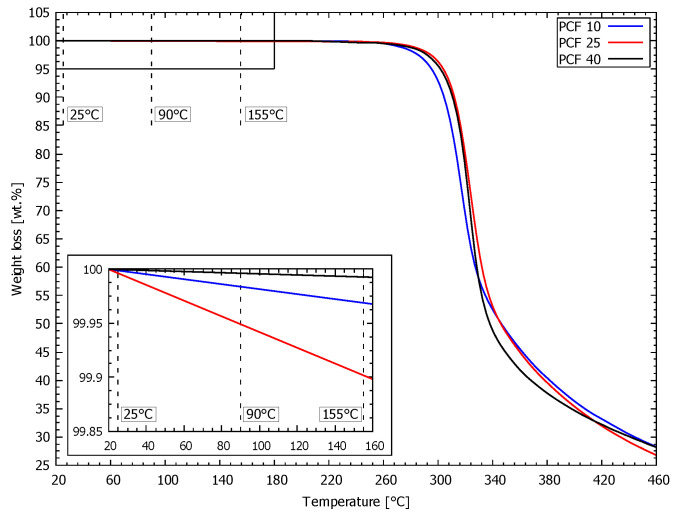
Thermogravimetric analysis (TGA) results of all analysed pure PUR foams.

The thermal properties of the analysed PUR foams were characterised by TGA. The TGA results in the form of percentage weight loss as a function of temperature are plotted in Figure 3. The value of weight loss at the analysis temperatures is presented in Table 3.

The weight losses at 25, 90 and 155 °C were subtracted from the thermogravimetric (TG) records (see Figure 3) because these temperatures were used as a reference during mechanical testing. The weight loss of all analysed materials is on the order of 0.1 wt% in the temperature interval from 25 °C to 155 °C. The greatest weight loss was observed for PUR foam with density PCF 25 at 155 °C (0.855 wt%), and the lowest value of weight loss was found for the sample with density PCF 40 at 155 °C (0.008 wt%). The weight loss for the sample with density PCF 10 at 155 °C was 0.009 wt%. The same trends were identified for other measurements at 25 °C and 90 °C.

### 3.3. Tension Tests

**Figure 4:**
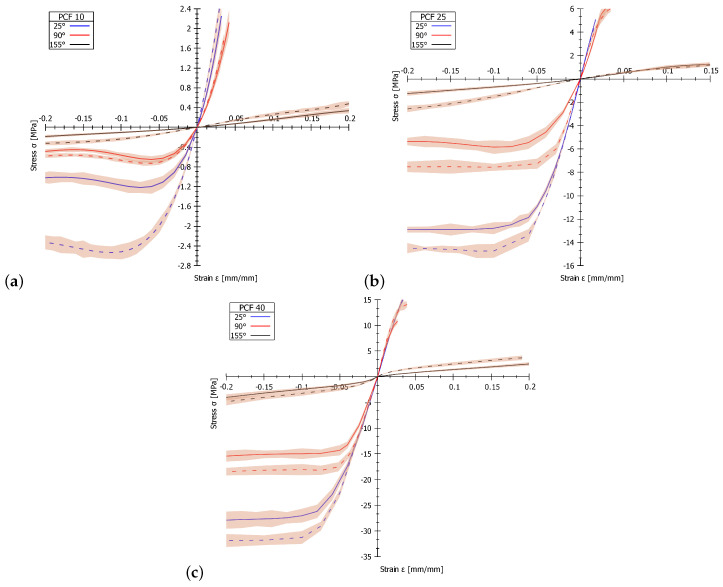
Stress–strain curves for tension and compression test of all analysed PUR foams: (**a**) PUR foam PCF 10, (**b**) PUR foam PCF 25 and (**c**) PUR foam PCF 40. Strain rate of 4.5 mm/min is the full lines, and strain rate of 45 mm/min is the dashed lines. The gray area is the range of the variance of the values.

The most common parameter describing technical materials in the linear region is Young’s modulus of elasticity. However, this parameter is insufficient to describe the mechanical properties of nonlinear materials such as PUR foam. A suitable way to describe nonlinear material is a complete description of the stress–strain dependence. These values can also be used as input parameters for finite element analyses (FEA). Figure 4 shows the stress–strain curves of the PUR foams under different environmental conditions. The values of the tensile modulus and tensile strength for all analysed PUR foams with different density, two strain rates (4.5 and 45 mm/min) and three temperatures (25 °C, 90 °C and 155 °C) are given in Table 4. The results of the experimental measurements were evaluated with a focus on the influence of temperature and strain rate of the tested samples.

From the results of the measurements at a strain rate of 4.5 mm/min, the tensile modulus and tensile strength decrease with increasing temperature in all tested PUR foam samples. The tensile modulus for the PUR foam with density PCF 10 decreases from 79.18 MPa at 25 °C up to 1.66 MPa at 155 °C. For the PUR foam with density PCF 25, the tensile modulus decreases from 297.37 MPa at 25 °C to 12.34 MPa at 155 °C. For PUR foam with density PCF 40, the tensile modulus decreases from 500.69 MPa at 25 °C to 17.02 MPa at 155 °C. A more detailed analysis of the results reveals that degradation of mechanical properties in the temperature range from 25 °C to 90 °C is relatively small for PCF 25 and PCF 40 (i.e., the tensile modulus for PCF 25 decreases by 6.1%, and that for PCF 40 decreases by 3.9%). When the temperature reaches 155 °C, the decrease of the tensile modulus drop is substantial (for PCF 25: 95.8%; for PCF 40: 97.0%). The mechanical properties of the PUR foam with density PCF 10 degrades almost linearly within the temperature range from 25 °C to 155 °C. The tensile strength values follow the same trend as the aforementioned tensile modulus values. Details of the tensile strength values are given in Table 4.

On the basis of the results of the experimental measurements presented in Table 4, even for a tenfold higher strain rate (45 mm/min), the behaviour of PUR foam at various temperatures is similar. The tensile modulus for PUR foam with density PCF 10 decreases from 86.25 MPa at 25 °C to 3.01 MPa at 155 °C. For the PUR foam with density PCF 25, the tensile modulus decreases from 309.06 MPa at 25 °C to 8.48 MPa at 155 °C. For PUR foam with density PCF 40, the tensile modulus decreases from 543.95 MPa at 25 °C up to 71.29 MPa at 155 °C. A more detailed analysis of the results reveals that degradation of mechanical properties in the temperature range from 25 °C to 90 °C is relatively small for PCF 25 and PCF 40 (the tensile modulus for PCF 25 decreases by 9.3%, and that for PCF 40 decreases by 6.8%). When the temperature reaches 155 °C, the decrease in tensile modulus is substantial (for PCF 25: 97.3%; for PCF 40: 86.9%). The PUR foam with density PCF 10 shows an almost linear degradation of mechanical properties within the temperature range from 25 °C to 155 °C. The tensile strength values follow the same trend as the aforementioned tensile modulus values. Detailed tensile strength values are given in Table 4.

The results of experimental measurements focusing on the strain rate reveal that, at temperatures ranging from 25 °C to 90 °C, the values of tensile modulus and tensile strength are almost identical in all analysed materials. Minor differences in the values of tensile modulus and tensile strength do not exceed the magnitude of the standard deviation; the tensile modulus and tensile strength can therefore be considered almost identical. The situation at the higher temperature of 155 °C is quite different: the values of tensile modulus and tensile strength of all tested materials increase with the increasing strain rate. At a strain rate of 45 mm/min, the tensile modulus value increases by 81.3% for PCF 10 and by 318.9% for PCF 40; however, for PCF 25, the tensile modulus decreases by 31.2%. The values of tensile strength at a strain rate of 45 mm/min are almost identical to those for a strain rate of 4.5 mm/min in all analysed materials. Minor differences in values of tensile strength do not exceed the magnitude of the standard deviation; the tensile strength values can therefore be considered almost identical.

### 3.4. Compression Tests

Figure 4 shows the mechanical behaviour of PUR foams, as obtained from stress–strain curves, under different environmental conditions. The values of the compressive modulus and compressive strength for all analysed PUR foams with different densities, as obtained at two strain rates (4.5 and 45 mm/min) and three temperatures (25 °C, 90 °C and 155 °C), are given in Table 5. The experimental measurements were evaluated with focus on the influence of temperature and strain rate on the properties of the tested samples.

The measurement results corresponding to a strain rate 4.5 mm/min clearly show that, with increasing temperature, the compressive modulus and compressive strength decrease in all of the tested PUR foam samples. The compressive modulus for the PUR foam with density PCF 10 decreases from 53.67 MPa at 25 °C to 0.94 MPa at 155 °C. For the PUR foam with density PCF 25, the compressive modulus decreases from 227.82 MPa at 25 °C to 6.38 MPa at 155 °C. For the PUR foam with density PCF 40, the compressive modulus decreases from 322.65 MPa at 25 °C to 42.38 MPa at 155 °C. A more detailed analysis of the results clarifies that the degradation of mechanical properties in the temperature range from 25 °C to 90 °C is relatively small for PCF 40: the compressive modulus decreases by 5.1%. When the temperature reaches 155 °C, the compressive modulus decreases by 86.9%. For both PCF 10 and PCF 25, the mechanical properties degrade almost immediately as the temperature increasea. For PCF 10, the value of the compressive modulus decreased by 15.7% at 90 °C and by 98.2% at 155 °C. For material PCF 25, the value of the compressive modulus decreases by 38.4% at 90 °C and by 97.2% at 155 °C. The compressive strength values decrease linearly with increasing temperature for all analysed materials. Detailed compressive strength values are given in Table 5.

According to the results of the experimental measurements presented in Table 5, for a tenfold increase in strain rate (45 mm/min), the behaviour of the PUR foam at the same temperature differs with tension loading. The mechanical properties degrade with increasing temperature, as shown by the tension loading results. For the PUR foams with density PCF 10 and PCF 25, the degradation of mechanical properties within the temperature range from 25 °C to 155 °C is almost linear. The compressive modulus for the PUR foam with density PCF 10 decreases from 49.71 MPa at 25 °C to 3.96 MPa at 155 °C. For the PUR foam with density PCF 25, the compressive modulus decreases from 262.89 MPa at 25 °C to 6.52 MPa at 155 °C. For both materials, PCF 10 and PCF 25 degraded the mechanical properties immediately as the temperature increased. For material PCF 10, the value of the compressive modulus decreases by 46.9% at 90 °C and by 92.0% at 155 °C. For material PCF 25, the value of the compressive modulus decreases by 22.5% at 90 °C and by 97.5% at 155 °C. A completely different situation occurs with the foam with density PCF 40. First, the compressive modulus increases from 258.16 MPa at 25 °C to 452.02 MPa (by 75.1%) at 90 °C and then decreases to 54.92 MPa (by 78.7%) at 155 °C. The compressive strength values decrease linearly with increasing temperature for all of the analysed materials. Detailed compressive strength values are given in Table 5.

When evaluating the results of experimental measurements focusing on the strain rate, the results show that values of compressive strength at a strain rate of 45 mm/min are almost identical to those for a strain rate of 4.5 mm/min in all analysed materials. Minor differences in values of compressive strength do not exceed the size of the standard deviation; they can therefore be considered almost identical.

## 4. Discussion

The thermal properties of all analysed PUR foams were characterised by thermogravimetric analysis. A standard procedure was used to perform TGA, in which the test samples were conditioned for 24 h at constant temperature and relative humidity before analysis. Concerning the chemical stability of the analyzed material, these conditions were optimal for maintaining the repeatability and validity of the obtained results. The protective atmosphere of nitrogen used did not react with the tested samples and could, therefore, not affect the measured values in any way. Likewise, the heating rate of 20 °C/min was chosen based on proven procedures for this type of material. The size of the tested samples was chosen concerning the size of the measuring device, its performance, and the ability to record data. None of the above parameters affected the results of the experimental measurements.

Standardised rigid polyurethane (PUR) foam blocks (Sawbones, USA) with a uniform structure and ±10% variation in density were used for all analyses. Therefore, the closed-cell foam conformed to standard ASTM F-1839-08 for surface roughness measurement used only one specimen for each density. All of the PUR foam samples were analysed by a digital microscope to determine their surface roughness parameters. The results correlate with the fact that an increase in density of the samples reduces their roughness. The PCF 10 samples contain larger pores in their structure than the PCF 40 samples, and this effect strongly influences the mechanical properties of the samples. Samples with a higher density appear more compatible; better mechanical properties are therefore expected.

Tensile and compression tests were realized for seven samples for each PUR foam density, temperature and strain rate. The measurements were realized for three different temperatures in the range of 20 °C to 155 °C, the magnitude of which corresponded to the experimentally determined temperatures arising during drilling into bone tissue (data from our own experiments and [13]). All measured values of stress–strain curves were averaged using an arithmetic mean, and the standard deviation was determined. Arithmetic averages for the limit values of material parameters were also determined. The number of samples was sufficient when the standard deviation (STD) of none of the measured values exceeded 12%. The measurements show that, for a strain rate of 4.5 mm/min, the tensile modulus and tensile strength decrease with increasing temperature in all of the tested PUR foam samples. In a more detailed analysis of the results, the degradation of mechanical properties in the temperature range from 25 °C to 90 °C is relatively small for PCF 25 and PCF 40. When the temperature reaches 155 °C, the tensile modulus drop is substantial. The tensile strength values follow the same trend as the tensile modulus values. The results of the experimental measurements show that, even when the strain rate is increased tenfold (to 45 mm/min), the behaviour of the PUR foam at various temperatures is similar. An evaluation of the results of experimental measurements focused on the strain rate reveals that, at temperatures ranging from 25 °C to 90 °C, the values of tensile modulus and tensile strength are almost identical in all analysed materials. The situation at the higher temperature of 155 °C is quite different: the value of the tensile modulus and tensile strength of all tested materials increase with increasing strain rate. The tensile strength values at a strain rate of 45 mm/min are almost identical to those at a strain rate of 4.5 mm/min for all of the analysed materials. An evaluation of the compression properties of PUR foam samples via measurements at a strain rate 4.5 mm/min reveals that, with increasing temperature, the compressive modulus and compressive strength decrease. The degradation of mechanical properties in the temperature range from 25 °C to 90 °C is relatively small for PCF 40, for which compressive modulus decreases by 5.1%. When the temperature reaches 155 °C, the compressive modulus decreases by 86.9%. Both PCF 10 and PCF 25 exhibited degraded mechanical properties immediately with an increase in temperature. The compressive strength values decrease linearly with increasing temperature for all analysed materials. For a tenfold higher strain rate (45 mm/min), the behaviour of PUR foam at the same temperature differs from that for tension loading. Degradation of mechanical properties occurs during an increase in temperature. For materials PCF 10 and 25, the material properties degrade immediately with increasing temperature. A completely different situation occurs with the PCF 40 material. The compressive modulus first increases from 258.16 MPa at 25 °C to 452.02 MPa (by 75.1%) at 90 °C and then decreases to 54.92 MPa (by 78.7%) at 155 °C. The compressive strength values decrease linearly with increasing temperature for all of the analysed materials.

## 5. Conclusions

Special drills used in surgical procedures are routinely tested on certified PUR foams according to the specifications detailed in ASTM F-1839-08. These tests produce temperatures at which the maximum temperature value detected does not exceed 180 °C. Testing confirmed that the PUR foam samples are thermally stable in the investigated temperature interval from 25 °C to 155 °C. The results show that decomposition of all foams occurs only when the temperature exceeds 260 °C. The magnitude of the weight loss is dependent on the applied temperature; however, a dependence on sample density was not observed in the range 25 °C–155 °C. Differences during thermal degradation occur at temperatures as high as 260 °C when the decomposition begins for the PCF 10 sample. With regard to thermal stability in the temperature range from 20 °C to 260 °C, all of the analysed materials are safe for these particular applications and provide valid results. To increase the possibility of using PUR foam in other industrial applications, enhanced thermal resistance is necessary. An interesting possibility is the incorporation of nanoparticles into PUR foam for stabilisation against decomposition [34].

PUR foam is used in practice for testing implants as well as surgical instruments. Due to the fact that a large amount of heat is generated during machining, it is evident that it is necessary to know the material properties of PUR foam even at higher temperatures. However, such material parameters have not yet been published anywhere. Therefore, experimental measurements were performed to determine PUR foam’s material parameters for testing medical devices and surgical tools depending on temperature, foam density, and strain rate. On the basis of experimental measurements and evaluations of the obtained values, we agree with the practice that PUR foam is a suitable material for mechanical testing of medical devices. PUR foam has similar mechanical properties to physiological bone tissue and can be used to validly perform experimental tests of medical devices (bone screw, plates, implants, etc.). The main conclusion of this paper is the fact that PUR foam has relatively good thermal and mechanical stability to 220 °C and, therefore, is also suitable for testing surgical tools that generate heat during machining. Finally, all measured material parameters were determined as dependent on temperature, density and strain rate and can be used as input parameters for numerical FEM analyzes. Such detailed and comprehensive information describing the thermal and mechanical properties has not been published anywhere for PUR foam.

## Figures and Tables

**Table 1:** Physical properties of polyurethane (PUR) foam samples.

Sample	Density (PCF)	Density (g/cm^3^)	Volume Fraction	Shore D Harness
PCF 10	10	0.16	0.14	15
PCF 25	25	0.40	0.34	43
PCF 40	40	0.64	0.54	65

**Table 2:** Surface roughness measurement results for the tested samples.

Density	$S_{a}$ (μm)	$S_{z}$ (μm)	$S_{q}$ (μm)
PCF 10	93.4±5.6	933.6±51.2	115.4±6.9
PCF 25	53.2±3.1	681.2±39.8	68.6±3.2
PCF 40	48.7±2.4	647.7±37.6	63.3±3.0

**Table 3:** Thermal stability results for all analysed PUR foams.

Density	Weight Loss (wt.%) at Temperature (°C)		
	25 °C	90 °C	155 °C
PCF 10	$0.035\times 10^{-3}$	$0.489\times 10^{-3}$	$0.942\times 10^{-3}$
PCF 25	$3.160\times 10^{-3}$	$44.20\times 10^{-3}$	$85.50\times 10^{-3}$
PCF 40	$0.031\times 10^{-3}$	$0.431\times 10^{-3}$	$0.832\times 10^{-3}$

**Table 4:** Summary of the tensile tests results obtained at a strain rate 4.5 mm/min and 45 mm/min.

Sample	25 °C		90 °C		155 °C	
	Tensile Modulus (MPa)	Tensile Strength (MPa)	Tensile Modulus (MPa)	Tensile Strength (MPa)	Tensile Modulus (MPa)	Tensile Strength (MPa)
	**Strain Rate 4.5 mm/min at Temperature (°C)**					
PCF 10	79.18±9.84	1.99±0.40	59.32±10.68	1.63±0.29	1.66±0.38	0.37±0.08
PCF 25	297.37±48.26	4.94±1.04	279.37±62.45	3.22±1.43	12.34±2.10	1.27±0.22
PCF 40	570.69±80.11	15.31±2.45	548.45±87.18	10.82±2.27	17.02±3.06	2.68±0.48
	**Strain Rate 45 mm/min at Temperature (°C)**					
PCF 10	86.25±13.29	2.17±0.83	53.07±5.84	1.40±0.64	3.01±0.57	0.46±0.32
PCF 25	309.06±32.54	4.52±0.93	280.32±40.05	3.03±1.34	8.48±1.10	1.47±0.73
PCF 40	543.95±54.40	15.94±1.22	507.19±85.08	14.07±1.087	71.29±11.41	3.70±0.97

**Table 5:** Summary of the compressive test results: strain rates 4.5 mm/min and 45 mm/min.

Sample	25 °C		90 °C		155 °C	
	Compressive Modulus (MPa)	Compressive Strength (MPa)	Compressive Modulus (MPa)	Compressive Strength (MPa)	Compressive Modulus (MPa)	Compressive Strength (MPa)
	**Strain Rate 4.5 mm/min at Temperature (°C)**					
PCF 10	53.67±10.73	1.44±0.29	45.22±8.14	0.44±0.08	0.94±0.20	0.34±0.04
PCF 25	227.82±47.84	13.51±2.43	140.36±26.67	5.45±0.82	6.38±1.09	1.82±0.47
PCF 40	322.65±51.62	29.23±7.60	306.04±81.07	14.83±3.12	42.38±7.63	6.36±1.14
	**Strain Rate 45 mm/min at Temperature (°C)**					
PCF 10	49.71±9.94	2.47±0.49	26.38±4.75	0.65±0.07	3.96±0.52	0.44±0.09
PCF 25	262.89±55.21	14.77±3.10	203.83±20.38	7.64±0.92	6.52±0.59	1.78±0.45
PCF 40	258.16±41.31	32.41±4.86	452.02±94.92	18.06±3.43	54.92±9.89	6.96±0.57
